# A real-time reverse transcriptase polymerase chain reaction for detection and quantification of Vesiculovirus

**DOI:** 10.1590/0074-02760150456

**Published:** 2016-06

**Authors:** Aline Lavado Tolardo, William Marciel de Souza, Marilia Farignoli Romeiro, Luiz Carlos Vieira, Luciano Kleber de Souza Luna, Dyana Alves Henriques, Jansen de Araujo, Carlos Eduardo Hassegawa Siqueira, Tatiana Elias Colombo, Victor Hugo Aquino, Benedito Antonio Lopes da Fonseca, Roberta Vieira de Morais Bronzoni, Maurício Lacerda Nogueira, Edison Luiz Durigon, Luiz Tadeu Moraes Figueiredo

**Affiliations:** 1 Universidade de São Paulo, Faculdade de Medicina de Ribeirão Preto, Centro de Pesquisa em Virologia, Ribeirão Preto, SP, Brasil; 2 Universidade de São Paulo, Instituto de Ciências Biomédicas, Laboratório de Virologia Clínica e Molecular, São Paulo, SP, Brasil; 3 Universidade Federal de Mato Grosso, Instituto de Ciências da Saúde, Centro Universitário de Sinop, Sinop, MT, Brasil; 4 Faculdade de Medicina de São José do Rio Preto, Laboratório de Pesquisa em Virologia, São José do Rio Preto, SP, Brasil; 5 Universidade de São Paulo, Faculdade de Ciências Farmacêuticas de Ribeirão Preto, Departamento de Análises Clínicas, Toxicológicas e Bromatológicas, Laboratório de Virologia, Ribeirão Preto, SP, Brasil

**Keywords:** Vesiculovirus, quantitative real-time RT-PCR, diagnosis of vesicular stomatitis, zoonotic virus

## Abstract

Vesiculoviruses (VSV) are zoonotic viruses that cause vesicular stomatitis disease in cattle, horses and pigs, as well as sporadic human cases of acute febrile illness. Therefore, diagnosis of VSV infections by reliable laboratory techniques is important to allow a proper case management and implementation of strategies for the containment of virus spread. We show here a sensitive and reproducible real-time reverse transcriptase polymerase chain reaction (RT-PCR) for detection and quantification of VSV. The assay was evaluated with arthropods and serum samples obtained from horses, cattle and patients with acute febrile disease. The real-time RT-PCR amplified the Piry, Carajas, Alagoas and Indiana Vesiculovirus at a melting temperature 81.02 ± 0.8ºC, and the sensitivity of assay was estimated in 10 RNA copies/mL to the Piry Vesiculovirus. The viral genome has been detected in samples of horses and cattle, but not detected in human sera or arthropods. Thus, this assay allows a preliminary differential diagnosis of VSV infections.

Vesiculovirus (VSV) genus belongs to *Rhabdoviridae* family and shares a common elongated bullet-like shape. VSV are enveloped with helical nucleocapsids containing a single strand, negative-sense RNA. VSV are enzootic and maintained in nature by not fully understood mechanisms (Lichty et al. 2004). Experimental studies indicate that mosquitoes and sand flies can transmit VSV among reservoir animals and eventually, to humans (Mead et al. 2000). Alternatively, VSV can be transmitted by infected animals through direct contact or fomites (Smith et al. 2009).

Currently, nine species of VSV are recognised by the International Committee on Taxonomy of Viruses (ICTV). From those, vesicular stomatitis Alagoas virus (VSAV), vesicular stomatitis New Jersey virus (VSNJV) and vesicular stomatitis Indiana virus (VSINV) are endemic in the Americas. The infection by these viruses produces the vesicular stomatitis, a disease of cattle, horses, sheep and pigs, which is characterised by fever, generation of vesicles in oral mucosa, skin, teat and coronary band. Vesicular stomatitis reduces the productivity of the animals, and it is economically important, thus requiring animal control by quarantines and trade barriers (Rodriguez 2002, Figueiredo 2011, Cargnelutti et al. 2014).

From June-August 2013, cases of vesicular stomatitis disease affecting horses and cattle were reported to the Veterinary Hospital of the Federal University of Campina Grande, Paraiba state, Brazil. These outbreaks occurred in the towns of Paulista, São Bento, Patos, in Paraiba state, and Umarizal, Rio Grande do Norte state. To confirm the laboratory diagnostic, the samples were subjected to reverse transcriptase polymerase chain reaction (RT-PCR) able to differentiate between the VSAV and VSINV (Pauszek et al. 2011). Thus, the virus found in the samples was the VSAV, which also was subsequently sequenced and deposited in GenBank (Cargnelutti et al. 2014).

The Chandipura (CHPV) and Isfahan vesiculovirus are arthropod-borne VSV have been associated with important cases of an acute febrile disease in humans (Tesh et al. 1977, Menghani et al. 2012). CHPV has also been reported to cause severe encephalitis in children in Asia (Rajasekharan et al. 2014). In South America, human infections by VSV are rarely reported. However, serological studies have reported a high prevalence (4-17.7%) of specific Piry vesiculovirus (PIRYV) neutralising antibodies in populations from different regions of Brazil (Pinheiro et al. 1974, Figueiredo et al. 1985, Tavares-Neto et al. 1990). In addition, human disease was reported in five cases, all related to laboratory accidents, with typical acute infectious viral disease (Pinheiro et al. 1974, Figueiredo et al. 1985, Souza et al., in press). In the 1960‘, PIRYV, Cocal and Maraba virus were isolated in Brazil from opossum (*Philander opossum*), mites (*Gigantolaelaps sp*) and sand flies (*Lutzomyia sp*), respectively. However, the importance of these viruses in human public health is practically unknown (Travassos da Rosa et al. 1984, Souza et al., in press).

The diagnosis of VSV infection has been done mostly based on serologic tests (ELISA - Enzyme-linked immunosorbent assay), virus neutralisation, and/or isolation (Afshar et al. 1993, Figueiredo 2011, Menghani et al. 2012). Despite being easily propagated in cell culture, VSV diagnostic techniques by neutralisation and virus isolation are time-consuming and labor intensive. In the last decades, the detection of the viral genome by molecular techniques has been successfully used (Bonutti & Figueiredo 2005,Hole et al. 2006, Wilson et al. 2009, Cargnelutti et al. 2014).

The development of real time RT-PCR allows the quantification of the final amplified product, allowing extrapolating the initial amount of target DNA in the sample. In contrast, most conventional PCR assays are only qualitative. Due to the exponential amplification of the DNA, any variation in the conventional technique during the amplification can lead to large variations in the amount of the final product amplified. The real-time RT-PCR allowed an increase in automatisation of reactions, reducing the risk of human error (Mackay 2004). Using this molecular approach, we show here an easy, sensitive, and reproducible real-time RT-PCR for detection and quantification of VSV.

## MATERIALS AND METHODS

*Primers design* - The sequences for the glycoprotein complete gene of vesiculoviruses were retrieved from GenBank of National Center for Biotechnology Information (NCBI) database. These sequences were from PIRYV (D26175), CHPV (NC_020805; GU212858), Perinet virus (NC_025394), VSIV (AF473864; EU849003; AM690337; AF473865; AF473866), VSAV (NC_025353), VSNJV (KC905171; KP202364; KM012169; JX121111; JX121112; JX121110; NC_024473; JX121104) and Carajas virus (KM205015). The sequences were aligned using MAFFT v7.158b (Katoh & Standley 2013) and conserved regions in the glycoprotein sequences were utilised to design primers using the Geneious v.8.0.1 program. However, the primers set was design to conserved region, the primers is predominantly based on PIRYV. The primers that amplify 222 base pairs (bp) are Piry-Forward: 5’-CAGGTGGTATGGRCCSAAATA-3’ (Position 3395 to 3415) and Piry-Reverse: 5’-ATCCAGTGACCTCTATAATCATC-3’, (Position 3616 to 3594), both primers based on coding sequence of PIRYV (GenBank No. KU178986). Primer sequences were compared to other nucleotide sequences deposited in the collection database and cross-reactivation with common human pathogens was not observed. Subsequently these sets of primers were synthesised (Sigma Aldrich, São Paulo, Brazil).

*Viruses* - The VSV used for this study were PIRYV, Carajas, VSAV and VSINV (Table I). The viruses were propagated in C6/36 *Aedes albopictus* cells, and maintained for 36 hours at 28ºC with Leibovitz’s-15 (L-15) medium supplemented with 10% heat-inactivated fetal bovine serum (FBS), 50 mg/mL of gentamicin and 2 mg/mL of amphotericin B (Vitrocell, Campinas, SP, Brazil).

**TABLE I:** Viruses used to development of real-time reverse transcriptase polymerase chain reaction for Vesiculovirus

Genus	Virus	Strain	T_M_ peak (ºC)	Quantify (RNA copies/mL)
Vesiculovirus	Piry	Be An 41191	81,5 ± 0,1	2,3 x 10^6^
Vesiculovirus	Carajas	Be An 411459	81,3 ± 0,0	1,4 x 10^6^
Vesiculovirus	Alagoas	Bn/64	81,8 ± 0,02	1,2 x 10^6^
Vesiculovirus	Indiana	BN/79	81,4 ± 0,04	1,8 x 10^5^
Alphavirus	Mayaro	BeAr 20290	72,4 ± 0,08	na
Flavivirus	Rocio	SPH 34675	70,3 ± 0,10	na
Orthobunyavirus	Oropouche	BeAn19991	75,3 ± 0,09	na

na: not amplified; T_M_: melting temperature.

*Viral RNA extraction and cDNA synthesis* - The RNA of viruses and samples were extracted using the QIAamp Viral RNA Mini Kit (Qiagen, Germany) according to the manufacturer’s protocol. The RNA was recovered in 60 μL of RNase-free water and stored at -70ºC.

For the reaction of cDNA synthesis, extracted RNA was reverse transcribed with 200U M-MLV (Invitrogen, Waltham, MA, USA), 10 μM random primers, 2.5 nM dNTPs, 0.1 M DTT, 20U RNAse-OUT (Invitrogen, Waltham, MA, USA) and RNase-free water. The reaction was incubated at 37ºC for 1 h, followed by enzyme inactivation at 70ºC for 15 min. The cDNA samples were stored at -20ºC until processed.

*Real-time PCR for Vesiculovirus* - The real-time PCR for VSV was performed in a StepOnePlus real-time PCR System (Applied Biosystems, Foster City, CA, USA). The reaction was standardised using the KAPA SYBR FAST Universal 2X qPCR Master Mix (Kapa Biosystems, Wilmington, MA, USA). The SYBR FAST Universal 2X qPCR Master Mix (Kapa Biosystems, Wilmington, MA, USA) was used in a reaction mixture including 2µL of cDNA template: 2 µL of each primer (Piry-Forward and Piry-Reverse at 2.5 mM per µL of all studied VSV; 0,4 µL of ROX buffer (2X); 10 µL of SYBR buffer (2X); and 3.6 µL of DEPC (Diethylpyrocarbonate) water, for a volume reaction of 20 µL. Different temperature cycles were also tested, and an optimal reaction was obtained with 95ºC for 10 min (to activate the Taq polymerase and separate double-stranded DNAs); and 45 cycles at 95ºC for 15 s for denaturation; 60ºC for 1 min for primer annealing.

*Plasmid cloning* - The amplicon of 222 nucleotides containing part of glycoprotein gene of PIRYV, obtained by RT-PCR with the selected primers, was cloned into the pET28a vector and introduced into *Escherichia coli* DH5α One Shot (Invitrogen, Waltham, MA, USA) following the manufacturer’s protocol. After transformation of competent cells with the insert, the plasmid DNA was isolated using the QIAprep Spin Miniprep Kit (Qiagen, Hilden, Germany).

*In vitro transcription* - A PCR was performed to amplify the 222 nucleotides of VSV inserted into the plasmid using Taq DNA polymerase (Invitrogen, Waltham, MA, USA), 5′-end primer containing T7 promoter region (5′-TAATACGACTCACTATAGGG-3′) and a T7 terminator region (5′-GCTAGTTATTGCTCAGCGG-3′), as recommended by the manufacturer. The cycling conditions were the following: 3 minutes for initial denaturation at 94ºC, followed by 45 cycles with 45 s at 94ºC for denaturation, 30 s at 58ºC for annealing and 90 s at 72ºC for extension. Finally, it was also used an extension of 5 min at 72ºC. The amplicon was purified using the QIAquick PCR Purification Kit (Qiagen, Hilden, Germany) and transcribed in vitro using a MEGAscript® T7 Transcription Kit (Invitrogen, Waltham, MA, USA), all following the manufacturer’s instructions. The reaction product was treated with 30U of Turbo DNAse-free (Invitrogen, Waltham, MA, USA), incubated 4 hours at 37ºC and inactivated at 70ºC for 15 min. Finally, it was performed the RNA purification using RNeasy kit (Qiagen, Hilden, Germany), the RNA load was determined in a Qubit® 2.0 Fluorometer (Invitrogen, Waltham, MA, USA) and the product was stored at -70ºC.

*Standard curve, detection limit and specificity* - The standard curve for VSV-RNA quantitation was obtained with serial ten-fold dilutions of the transcribed RNA. The assay was performed in triplicate and the concentration was measured in copies per mL, was converted to copy number using the following formula: RNA copy number (copies/mL) = (RNA concentration (g/mL)/number of nucleotides of transcript × 340) × 6.022 × 10^23^. For each new reaction, was obtained a standard curve based on the results obtained from the serial dilutions of the transcribed product.

*Samples* - *Human sera* - A total of 410 sera from patients with acute febrile illnesses were tested by the real-time RT-PCR for VSV. Dengue virus was not previously detected by conventional RT-PCR in these samples (Bronzoni et al. 2005). The sera included 88 samples from patients obtained during an outbreak of Dengue virus, 2006-2014, at São José do Rio Preto city (155 samples), Ribeirão Preto city (188 samples), both in São Paulo state, Brazil, and 67 samples from Sinop city, Mato Grosso state, Brazil. This study was approved by the Human Research Ethics Committee, School of Medicine of University of São Paulo, Brazil (Process No. 2013/164.277).

*Arthropod pools* - Seventy-six lots of arthropods were tested by real-time RT-PCR to detect Vesiculovirus in potential vectors. The samples included: 57 pools of different species of mosquitos, collected in 2007, in different places of Rondônia state, Brazil. Also, were included 19 pools of ticks collected in domestic animals in 2014, in Ribeirão Preto city, São Paulo state (10 pools of*Rhipichephalus sp* and 9 pools of *Amblyomma sp*). The samples of arthropods were disrupted and homogenised with TissueLyser II (Qiagen, Hilden, Germany). The RNA of all samples was extracted using the QIAamp Viral RNA Mini Kit (Qiagen, Hilden, Germany) and cDNAs were obtained as described above.

*Cattle and horses* - The real-time RT-PCR for VSV was also used to test serum samples from 18 cattle and horses obtained during an outbreak of VSV in towns of northeastern of Brazil (Cargnelutti et al. 2014). These samples were kindly provided by Prof Dr Eduardo Furtado Flores and Dr Juliana Cargnelutti, from the Federal University of Santa Maria, Rio Grande do Sul, Brazil.

## RESULTS

**Fig. 1:**
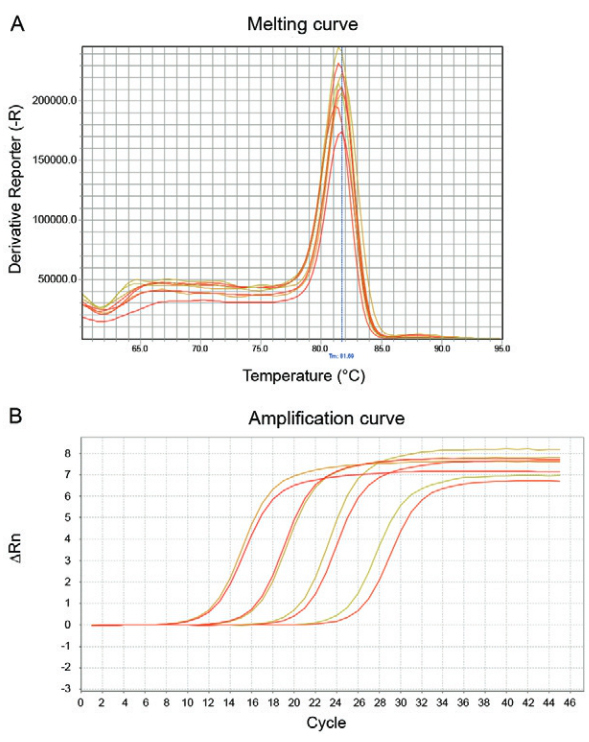
: standard line of real-time reverse transcriptase polymerase chain reaction (RT-PCR) with transcribed RNA of Vesiculovirus. (A) Standard line with serial decimal dilutions of transcribed RNA. (B) Amplification curve obtained from of serial decimal dilutions of transcribed RNA. (C) Melting peaks of real-time RT-PCR of serial decimal dilutions of the transcribed RNA.

The parameters obtained with decimal dilutions of the in vitro transcribed RNA for the standard curve of the quantitative SYBR Green real-time RTPCR are the following: slope -3.036, percentage efficiency (EFF) 113.508%; correlation coefficient (R_2_) 0.978 and Y-inter 39.28. The threshold value of 1.636 for the RNA dilutions was reproducible with a T_M_ (melting temperature) peak of 81.06ºC to 82.1ºC (Fig. 1).

**Fig. 2:**
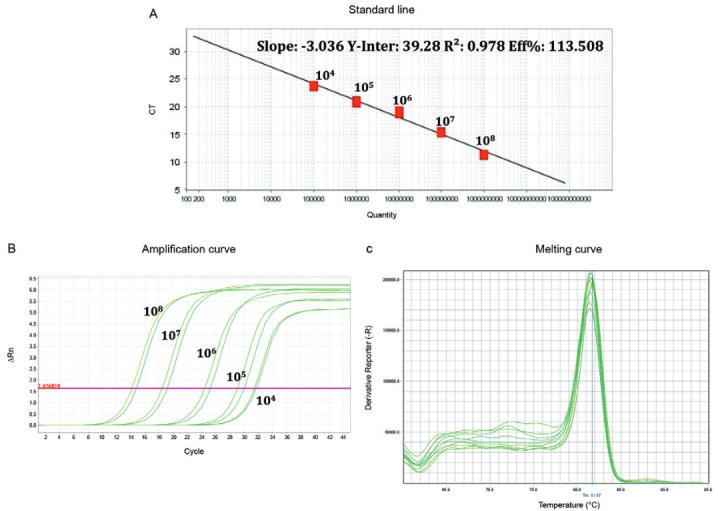
: melting peaks and amplification curve of real-time reverse transcriptase polymerase chain reaction (RT-PCR) for Vesiculovirus. (A) Melting peaks of real-time RT-PCR obtained from Vesiculovirus described in Table I. (B) Amplification curve obtained for the Vesiculovirus described in Table I.

The real-time RT-PCR showed a sensitivity of 10 copies/mL for all the VSV tested (Alagoas, Carajas, PIRYV and VSINV) and specific T_M_ peaks without the formation of primer-dimer for all of them, as shown in Table I and Fig. 2. Besides, the genomes of *Flavivirus* (Rocio strain SPH 34675, Cacipacore strain BeAn 327600 and Ilheus virus strain BeH 7445) and*Orthobunyavirus* (Oropouche strain BeAn19991), used as negative controls, were not amplified in the test.

The real-time RT-PCR was able to detect the virus genome in 15 of 17 serum samples from cattle and horses obtained during an outbreak of Alagoas virus. There was no significant difference between VSV loads observed in horses (1.5 x 10^1^ to 2 x 10^6^ RNA copies/mL) and those from bovines (1.4 x 10^1^ to 1.2 x 10^5^ RNA copies/mL), as shown in Table II. Unfortunately, the VSV genome was not detected in human sera, as well as in the arthropods analysed in the study.

**TABLE II:** Samples positive found in this study by real-time reverse transcriptase polymerase chain reaction for vesicular stomatitis Alagoas virus

Samples^1^	T_M_ (ºC)	Viral load (RNA copies/mL)
01 Equine	81,3 ± 0,00*	1,2 x 10^6^
02 Equine	81,1 ± 0,10	1,3 x 10^4^
03 Equine	81,1 ± 0,00*	1,9 x 10^1^
04 Equine	81,2 ± 0,10	2,6 x 10^1^
05 Equine	81,4 ± 0,09	1,3 x 10^4^
06 Equine	81,02 ± 0,10	2,3 x 10^4^
07 Equine	81,1 ± 0,00*	1,5 x 10^1^
08 Equine	81,7 ± 0,08	1,5 x 10^1^
09 Equine	81,3 ± 0,07	5,5 x 10^2^
10 Equine	81,1 ± 0,20	5,7 x 10^2^
01 Bovine	81,2 ± 0,10	1,2 x 10^5^
02 Bovine	81,4 ± 0,09	2,1 x 10^2^
03 Bovine	81,7 ± 0,00*	8,3 x 10^3^
04 Bovine	81,6 ± 0,20	1,4 x 10^1^
05 Bovine	81,5 ± 0,04	9,1 x 10^3^

T_M_: melting temperature; *Standard deviation less than 0.001; ^1^ Cargnelutti et al. (2014).

## DISCUSSION

VSV detection by laboratory tests such as virus isolation (gold standard), serologic tests, or neutralisation assays is time-consuming. Additionally, the available conventional PCR assays usually detect few species VSV and an extra electrophoresis step is required to visualise the amplificons (Rodriguez et al. 1993, Fernández et al. 2008). Therefore, we decided to develop a real-time RT-PCR assay to detect a broad range of VSV species that could also be used for epidemiological surveillance.

Molecular techniques have been previously used for VSV diagnosis, as well as probes has been developed for differentiation of VSNJV and VSINV (Hole et al. 2006) or for detection and quantification of CHPV (Kumar et al. 2008). To the best of our knowledge, no real-time RT-PCR using SYBR Green methodology has been reported for detection of VSV.

The real-time RT-PCR for VSV was set using the SYBR Green I method and including specially designed primers that anneal in highly conserved region of the glycoprotein of VSV. The primers were able to amplify the genomes of four taxonomically distinct VSV: VSINV (Indiana 1 group), VSAV (Indiana 3 group), Carajas virus (isolated from an insect and taxonomically closer related to VSNJV) and PIRYV (South American zoonotic virus). Therefore, based on the primer design and on our results, it is strongly probable that this SYBR Green I real-time RT-PCR can be used to detect, practically, all American VSV.

The lower cost compared to TaqMan technique, coupled with the fact that we developed a generic PCR, led us to the choice of SYBR Green real-time method. Although less specific than TaqMan, SYBR Green allowed a specific generic identification of the VSV, since this technique has amplify the four virus specifically and efficiently and did not amplify other viruses tested, for example,*Orthobunyavirus*, *Alphavirus* and*Flavivirus*. The advantages this assay are: (i) low cost, (ii) capable to detect and quantify VSVs, (iii) binding of SYBR Green to nucleic acid is not virus specific and the fluorescent signal produced, when in complex with DNA, is precise and directly proportional to the length and the amount of DNA copies synthesised (Papin et al. 2004).

The real-time RT-PCR was highly sensitive with a limit of detection of 10 copies of RNA per mL. Also, this assay has been showed that is genus-specific because primer dimers or unspecific amplification were not observed and crossamplification with genomes of other arbovirus such as Orthobunyavirus, Alphavirus, and Flavivirus, did not occur. We report a percentage efficiency (EFF) was higher than 100%. This value is within the accepted range (90-120%) recommended by the manufacturer. Also, the efficiency of the assays is supported by appropriate R2 and slope values. Also, the amplicons were visualised by electrophoresis (Data not shown).

The test was able to detect VSAV with variable titers in 15 of 18 sera from cattle and horses that were obtained during an outbreak, previously reported in northeastern Brazil (Cargnelutti et al. 2014). On the other hand, we believe that improper storage of samples at -20ºC resulted in a decrease in viral loads, including low VSAV titers and non-detection of the genome into three sera samples. The samples were previously sequenced by Cargnelutti et al. (2014). This demonstrates that this assay can be useful for VSV diagnosis of animals, especially since the acute phase of infection by VSV generally have high titers (Schmitt 2002).

The serosurveys studies performed in several places of Brazil have shown neutralising antibodies to PIRYV in more than 10% of the participants. Based on an accidental infection in the laboratory, we know that PIRYV produces an acute febrile illness (Pinheiro et al. 1974, Figueiredo et al. 1985, Tavares-Neto et al. 1990, Souza et al., in press). In contrast, we have tested 410 sera of patients with acute febrile illness by the real-time RT-PCR for VSV, but all samples were negative for VSV. It is possible that most human infections by VSV in nature are asymptomatic, oligosymptomatic or produce a short period of viraemia. In addition, currently, little is known about mechanisms of maintenance and transmission of VSV in nature. Here, we have analysed ticks and mosquitoes from different places of Brazil, but all samples were negative to VSV. A novel, rapid, sensitive, VSV genus-specific, and reproducible SYBR Green real time RT-PCR was developed for detection and quantification of Vesiculovirus. This reaction was sensitive with a detection limit of 10 RNA copies/mL and proved to be able to diagnose and quantify VSV Alagoas in serum samples from cattle and equines.
